# 

**Published:** 1892-12

**Authors:** 


### CONTENTS.

*EDITORIAL.*

PAGE

The End of the Year, . . . . . . . . . . . . . . . 529

*MISCELLANEOUS.*

International Hahnemannian Association Meeting of 1892.

Fragmentary Proving of Potassium Chlorate. E. Rushmore, M. D., . . . . . . . . . . . . . 530

A Silica Symptom Verified. E. Rushmore, M. D., . . . . 530

Verifications of Medorrhinum. J. H. Allen, M. D., . . . 531

Note, . . . . . . . . . . . . . . . 532

Cereus Bonplandii. J. H. Fitch, M. D., . . . . . . 533

Bureau of Surgery. T. M. Dillingham, M. D., . . . . 543

Three Surgical Cases. Erastus E. Case, M. D., . . . . 543

Abscesses of the Vulva Cured by Sepia. I. Dever, M. D., . 548

Nasal Polypi Cured with One Dose of Aurum-m$^{50}$m. J. R. Haynes, M. D., . . . . . . . . . . . 551

The Institute Bureau of Materia Medica, . . . . . . 552

Graphites *vs.* Cicatricial Tissue. Caroline E. Hastings, M. D., 553

Necrosis of the Os Calcis. G. W. Sherbino, M. D., . . . . 555

Infantile Spinal Atrophic Paralysis. T. M. Dillingham, M. D., . . . . . . . . . . . . . 564

Poliomyelitis Anterior Acuta (Acute Inflammation of the Anterior Gray Horns, Acute Atrophic Spinal Paralysis, . 568

Infantile Spinal Atrophic Paralysis, . . . . . . . . 568

Removal of Ovaries, . . . . . . . . . . . . 582

*BOOK NOTICES.*

The Physician's Visiting List for 1893, . . . . . . . . 584

All Around the Year 1893, . . . . . . . . . . 584

